# The glomerular permeability factors in idiopathic nephrotic syndrome

**DOI:** 10.1007/s00467-015-3082-x

**Published:** 2015-04-30

**Authors:** Jean-Claude Davin

**Affiliations:** 1 Emma Children’s Hospital/ Academic Medical Centre, University of Amsterdam, Amsterdam, The Netherlands; 2 Queen Fabiola Academic Children’s Hospital, Free University of Brussels, Brussels, Belgium; 3 Pediatric Nephrology Department, Emma Children’s Hospital/ Academic Medical Centre, Meibergdreef 9, 1105 AZ Amsterdam, The Netherlands

**Keywords:** Circulating factors, Podocyte, Nephrotic syndrome, Glomerular permeability factors, Proteinuria

## Abstract

It is currently postulated that steroid-sensitive idiopathic nephrotic syndrome (SSNS) and steroid-resistant idiopathic nephrotic syndrome (SRNS), which are not related to the mutation of a gene coding for podocyte structures or for glomerular basement membrane proteins, result from a circulating factor affecting podocyte shape and function. T lymphocytes have for a long time been suspected to be involved in the pathophysiology of these diseases. The successful treatment of steroid-dependant nephrotic syndrome with rituximab suggests a potential role for B lymphocytes. Clinical and experimental data indicate roles for cytokines IL-13, TNFα, circulating cardiotrophin-like cytokine factor 1 (member of the IL-6 family), circulating hemopexin, radical oxygen species, and the soluble urokinase-type plasminogen activator receptor (suPAR) in the development of nephrotic syndrome. Podocyte metabolism modifications—leading to the overexpression of the podocyte B7-1antigen (CD 80), hypoactivity of the podocyte enzyme sphingomyelin phosphodiesterase acid-like 3 b (SMPDL3b), and to the podocyte production of a hyposialylated form of the angiopoietin-like 4 (Angptl4)—are mechanisms possibly involved in the changes in the podocyte cytoskeleton leading to SSNS and or SRNS. Different multifactorial pathophysiological mechanisms can be advocated for SSNS and SRNS. The present paper reviews the experimental and clinical data upon which the different hypotheses are based and reports their possible clinical applications.

## Introduction

Idiopathic nephrotic syndrome (INS) is the most frequent form of NS in children, representing more than 90 % of cases between 1 and 10 years of age and 50 % after 10 years of age [1]. INS is defined by the association of the clinical features of NS with renal biopsy findings of diffuse foot process effacement on electron microscopy and minimal changes (also called minimal change disease (MCD), or focal segmental glomerulosclerosis (FSGS), or diffuse mesangial proliferation (DMP) on light microscopy [2]. Most patients have histologic findings of MCD. The response to steroid therapy (steroid-sensitive nephrotic syndrome [SSNS] versus steroid-resistant nephrotic syndrome [SRNS]) is of higher prognostic significance than histologic features seen on initial renal biopsy [3]. Overall, the renal outcome of children with steroid-dependent NS is excellent as long as patients remain steroid responsive [3]. Despite a favorable long-term outcome, adverse effects of treatment contribute to inferior quality of life of children and their families in case of SSNS [4]. The vast majority of patients with MCD (>90 %) respond to glucocorticoid therapy, whereas only 50 % of those with DMP and 30 % of those with FSGS are expected to do so [5]. Contrary to SSNS, SRNS leads to chronic kidney disease (CKD) and end-stage renal failure (ESRF) in about 50 % of patients [6, 7]. In a recent study, a single-gene cause was found in 29.5 % of cases of SRNS [8]. Within clinically relevant age groups, the fraction of families with detection of the single-gene cause was as follows: onset in the first 3 months of life (69.4 %), between 4 and 12 months old (49.7 %), between 1 and 6 years old (25.3 %), between 7 and 12 years old (17.8 %), and between 13 and 18 years old (10.8 %) [8].

In a cohort of sporadic SRNS (exhibiting no extra-renal symptoms, no familial history of the disease or consanguinity, and no congenital onset) a genetic cause was found in 32.3 % of the children with SRNS versus 0 % in 38 children with SSNS used as controls [9]. In the same study, genetic alterations were also associated with response to immunosuppressive agents in children with SRNS (0 % of patients with alterations responded versus 57.9 % of patients without alterations) [9]. Amazingly, however, some patients presenting with a mutation are steroid sensitive [10] or improve under cyclosporine [11, 12]. This apparently paradoxical response to cyclosporine might be attributed to the stabilization of the podocyte cytoskeleton by this compound [13].

### Evidence of a circulating factor in INS

The pathophysiological role of a circulating factor affecting the podocyte structure and function is mainly supported by the following observations: (1) nephrotic proteinuria healed spontaneously in a few days in a child born from a mother with FSGS and nephrotic syndrome [14]; (2) the successful transplantation in a diabetic patient of a graft removed from a transplanted FSGS recipient because of intractable recurrence of massive proteinuria and renal insufficiency [15]; (3) the perfusion of isolated rat glomeruli with plasma of patients with FSGS induces an increased glomerular capillary permeability to albumin as compared to normal control plasma [16]; (4) after kidney transplantation of an organ from a donor without the disease, approximately 30 % of patients with FSGS develop massive proteinuria within hours to days after transplantation followed later on by FSGS histological lesions [17]; (5) some of those patients are successfully treated with plasma exchange when applied early [17]; (6) preemptive plasmapheresis reduces the risk of FSGS recurrence after transplantation [18].

The vast majority of recurrences are not associated with mutations. Although exceptional, relapses of nephrotic syndrome after transplantation in some patients with mutations have been described (for a review, see 12). Some of them might be attributed to auto-antibodies directed to the neo-antigen constituted by the proteins of transplanted podocytes as in the case of defects for *NPHS1* gene [19]. However, this hypothesis is not applicable in case of recurrence when mutations concern antigens “hidden” within the cytoskeleton as podocin or actinin 4 [12].

## Immune response and permeability factors

Research on immunological causes of INS is complicated by the need to observe homogeneous patients groups, which is often not the case since the duration of proteinuria varies at the time of presentation and some patients may have already started treatment. Hyperlipidemia, which is a common complication, might modulate the immune system [20]. Those pitfalls may explain at least partly the discrepancies observed between the data of different studies.

The role of the immune system in the pathophysiology of INS has been suspected for decades. This hypothesis was suggested by the response to immunosuppressive drugs and the association with Hodgkin’s disease and with allergies [21]. Many reports have been published on patients who developed NS after having experienced allergic reactions to inhaled or ingested allergens after vaccinations and insect stings. Furthermore, the incidence of atopy was reported higher in patients with INS than in healthy subjects, ranging from 17 to 40 % in MCD patients compared with 10–23 % in age-matched control subjects (for a review, see 22). Allergies are associated with an elevated production of IgE by B-lymphocytes, and several investigators have reported an elevation of IgE in the serum of NS patients (for a review, see 22).

### T cells

The first experimental evidence suggesting a pathophysiological role of lymphocytes in INS was reported by Lagrue and co-workers in 1975 who showed that the supernatant of lymphocytes from patients with MCD stimulated in vitro by concanavalin A contains a factor that modifies the vascular permeability [23].

CD8-positive T cells of INS patients are clonally expanded, which is not observed in healthy controls [24]. High levels of NF-*κ*B (nuclear factor *κ*B) DNA-binding activity are observed in T cells from untreated MCD patients during relapse compared with the MCD patients in remission while treated with immunosuppressants [25]. In one in vitro study, T cells produced interleukin IL-13 spontaneously, and B cells constitutively expressed IL-13 receptors (IL-13R) [26]. In addition, T cells stimulated surface IgE-negative (sIgE-) and sIgG4- B cells to produce IgE and IgG4, respectively, and IgE and IgG4 production was specifically blocked by anti-IL-13 antibody [26]. An elevated expression of IL-13 mRNA was also shown [27]. Van de Berg and Weening [22] have studied, by quantitative real-time PCR, the expression of IL-1*β*, IL-1ra (IL-1 receptor antagonist), IL-2, IL-4, IL-5, IL-9, IL-10, IL-13, TNF-*α*, and IFN-*γ* by PBMC from patients with MCD during relapse and remission and from a control group of patients with NS primarily caused by endogenous alterations within the glomerular filter, for instance, mutations in the genes encoding nephrin and podocin. Out of the cytokines studied, only the expression of IL-10 and IL-13 mRNA was significantly up-regulated in relapsing MCD patients when compared with MCD patients in remission. The latter authors and others (for a review, see 22) have shown that podocytes constitutively express functional trans-membrane receptor complexes for IL-4, IL-10, IL-13, and TNF-*α*. The possible role of IL-13 is also suggested by a rat model of NS [28]. IL-13 was overexpressed in Wistar rats through transfection of a mammalian expression vector cloned with the rat IL-13 gene. The IL-13-transfected rats showed significant albuminuria, hypoalbuminemia, and hypercholesterolemia. No significant histologic changes were seen in glomeruli. However, electron microscopy showed up to 80 % of podocyte’s foot process fusion. Glomerular gene expression was significantly up-regulated for CD80, IL 4R, and IL13R and downregulated for nephrin, podocin, and dystroglycan. Immunofluorescence staining intensity was reduced for nephrin, podocin, and dystroglycan [28].

Because of the pathogenetic role of IL-13 in asthma and the induction of glomerular CD80 gene expression in an IL-13 induced experimental model of proteinuria, it has been suggested that the relation between allergy and INS could be the stimulation by IL-13 of the expression of CD80 on fragile podocytes [29]. Urinary CD80 levels are increased in patients with MCD during relapse and return to normal after remission [30]. Evidence that the source of the CD80 is the podocyte was suggested by the finding that CD80 was expressed by podocytes in kidney biopsy specimens from patients with MCD in relapse and that urinary CD80 molecular weight was compatible with a podocyte origin [30].

TNF alpha is secreted by T cells and other types of leucocytes. The successful treatment of SRNS on native kidney or of SRNS relapse after kidney transplantation with anti-TNF*α* antibodies strongly suggests that TNF*α* participates to the pathogenesis of some types of INS [31, 32]. This hypothesis is also supported by high blood levels of TNF*α* in patients with active disease, normalizing with remission and by an animal model of NS that is controlled by anti-TNF*α* agents (for a review, see 31, 32). Bitzan et al. have shown that podocyte β3-integrin can be activated by plasma from patients with FSGS recurrence and this activation could be reversed by blocking the TNF-α pathway [33].

### B cells

The beneficial treatment by rituximab, a monoclonal antibody directed against CD20, in difficult SSNSs suggests a pathophysiological role for B cells [34–37]. A recent systematic review of 39 reported cases (from whom 19 were pediatric) of FSGS recurrence on kidney transplant treated with rituximab showed that complete remission occurred in 43.5 % of patients [38]. Multivariate analysis revealed that normal serum albumin at FSGS recurrence and lower age at transplant were associated with response [38]. B cells may be involved through an unidentified antibody-independent pathway, which might be a control on T cells [39]. However, B cells might act more directly. Indeed, observations of MCD in pathologies associated with monoclonal light chains highly suggest a potential implication of immunoglobulins and/or of defective machinery leading to abnormal immunoglobulins [40]. Other arguments for a B cell role are: the detection of immunoglobulin in glomeruli from half the patients (for review, see 41), the prevention of relapses during B cell depletion in a majority of patients treated with rituximab [36, 37], the concept of IgM nephropathy and the demonstration in a subgroup of patients with INS of circulating antibodies against actin, a major molecule of a podocyte’s cytoskeleton [42]. A significant association of HLA-DQA1 (a MHC class II) missense coding variants with SSNS recently brought a confirmation of the possible role of an immune response and of the implication of B cells in the pathogenesis of this disease [43].

### Circulating cardiotrophin-like cytokine factor 1

The group of Virginia Savin in US has studied and characterized the circulating factor in FSGS by analyzing the plasma of patients presenting with a post-transplant relapse (for a review, see 16). Those studies are based on standard methods of biochemical purification and analyses of molecular characteristics followed by gel electrophoresis and mass spectrometry. They have used a functional assay of permeability activity with isolated rat glomeruli that shows changes in the glomerular capillary permeability to albumin after incubation with the patient plasma or serum. This assay has made it possible to perform sequential purification steps and select fraction(s) with enhanced activity. They have shown that the focal sclerosis permeability factor (FSPF) resides in a 30- to 50-kDa plasma fraction. Using galactose as an effective affinity material to enrich activity of FSGS plasma, they reported that cardiotrophin-like cytokine factor 1 (CLC-1; encoded by *CLCF1*), a member of the interleukin 6 family, is present in this enriched fraction of FSGS plasma. CLC-1 increases glomerular Palb and its injection causes proteinuria in rats. However, those experiments do not exclude the involvement of other galactose-binding permeability factors. Interestingly, CLC-1, may be obtained from activated T cells in vitro and is able to stimulate B cells [44]. Those authors hypothesized that galactose administered orally or intravenously at an early stage might prevent the development of CKD in patients with FSGS by impeding the binding of the circulating factor on galactose residues present at the surface of podocytes [45, 46]. The therapeutic use of galactose is actually under investigation in clinical trials [47]. A pilot study on this topic was recently published [48]. Seven pediatric subjects with idiopathic SRNS and positive FSPF activity were treated with oral galactose (0.2 g/kg/dose twice daily) for 16 weeks [48]. The treatment induced a reduction of FSPF activity but not of proteinuria. It has been argued that this lack of response might have been due to the already constituted FSGS lesions at the moment of treatment and that galactose is expected to be protective only in an early phase of the disease [49].

## Radical oxygen species (ROS)

Some experimental models of NS are obtained using substances as puromycin and adriamycin that induce oxidative stress in glomeruli (for a review, see 50). Furthermore, the injection of H_2_O_2_ induces proteinuria in rats and NO prevents the increase of permeability to albumin induced by the TNF alpha-induced O_2_- production in an isolated rat glomeruli system [51]. Active FSGS is associated with massive oxidation of plasma albumin [50]. Bertelli et al. [52] demonstrated a tenfold increase of ROS production by resting PMN from INS patients compared to normal PMN. Those authors have also shown that the oxidative burst by PMN was regulated highly by T lymphocytes, mainly Tregs, by means of soluble factors and that this regulatory circuit was altered in INS [52].

## Hemopexin

It has been shown that nephrotic plasma alters a slit diaphragm-dependent signaling and translocates nephrin, podocin, and CD2 associated protein in cultured human podocytes [53]. This alteration might be due to hemopexin (Hx) [54]. Hx is a heme-scavenging protein. It is predominantly produced in the liver, and it increases in the acute phase reaction to inflammation or infection. Plasma-purified and recombinant Hx has been shown to have serine protease activity [54]. It has been suggested that in normal conditions, circulating Hx is inactive but under certain circumstances Hx becomes activated as a serine protease [54]. Activated Hx has been shown to have dramatic effects on the glomerular filtration barrier. Kidney sections incubated with Hx have a reduction of the anionic layer and of sialoglycoproteins [55]. In vivo, activated Hx induced reversible proteinuria in rats parallel to podocyte foot process effacement [54]. Activated hemopexin is increased in children during MCD relapses [54]. After in vitro treatment with hemopexin, actin reorganized from stress fibers to cytoplasmic aggregates and membrane ruffles in wild-type podocytes [55]. This process is nephrin-dependent since it did not occur in nephrin-deficient podocytes and in cells that do not express nephrin and was inhibited by preincubation with normal human plasma. In addition, hemopexin led to a selective increase in the passage of albumin across monolayers of glomerular endothelial cells and to a reduction in glycocalyx [55]. What remains to be elucidated is the primary events leading to the activation of Hx. A possibility resides in the inhibition of Hx inhibitors or in their leakage in urine. In the latter case, Hx activation should be only a secondary event depending on the increased permeability of the glomerular filtration barrier to proteins.

## Soluble urokinase-type plasminogen activator receptor (su-PAR)

The eventual role of urokinase-type plasminogen activator receptor (uPAR) in its soluble form (suPAR) in the pathogenesis of FSGS in human is actually in the center of a debate between scientists. A review on this topic has been recently published in Pediatric Nephrology [56].

The initializing event of FSGS seems to be the migration along the glomerular basement membrane and the detachment of podocytes. uPAR has a role in the migration of activated T lymphocytes, monocytes, and neutrophils to sites of inflammation (for a review, see 56). Overexpression of uPAR is associated with disease progression in malignancies (see 56). Those observations led to setting up experimental models of FSGS using suPAR.

The biochemical features of uPAR are as follows [56]: glycosylphosphatidylinositol (GPI)-anchored membrane glycoprotein; consists of three homologous domains (DI, DII and DIII), which are encoded in PLAUR gene; molecular mass between 35 and 60 kDa depending on the glycosylated state, whereas suPAR molecular weight ranges from 20 to 50 kD. Of importance, those MW ranges are similar to that found for the FSPF by the group of Savin [16].

Podocyte foot processes contain an actin-based cytoskeleton that is linked to the vitronectin molecules of the glomerular basement membrane by a3ß1 and avß3 integrin, which bind to vitronectin (for a review, see 56). The stimulation of integrins induces intracellular processes leading to modifications of the actin cytoskeleton. Podocyte’s uPAR binds to integrin and to vitronectin. In podocyte cultures and murine models, uPAR was shown to cause vitronectin-dependent αvβ3-integrin activation [57].

The hypothesis that suPAR induces FSGS in mice is sustained by the fact that high-dose recombinant mouse suPARI-III induced proteinuria in PLAUR knockout mice (missing suPAR), indicating that circulating suPAR may activate β3-integrin independent of uPAR [57].

Indirect experimental observations using the plasma of patients with FSGS suggest the pathogenetic role of suPAR in humans also (for a review, see 56).

Increased activation of β3-integrin was observed when differentiated podocytes were exposed to sera from patients with recurrent FSGS. Podocyte β3-integrin activation was reduced when sera from patients with complete proteinuria remission after plasmapheresis were used, and it was blocked by antibodies against uPAR and by a β3-integrin inhibitor. Increased β3-integrin activation was also observed in the glomeruli of patients with primary FSGS in their native or transplant kidney, compared to controls with minimal change disease or membranous nephropathy.

Two studies suggested that serum suPAR was significantly increased in patients with FSGS as compared to patients with other glomerular diseases inclusively MCD [58, 59], Importantly, in those two studies, the serum suPAR data were not corrected for eGFR and several other studies failed to show any difference between suPAR in FSGS and controls when suPAR values were corrected for GFR [50–64].

In conclusion, taken together, the data obtained in clinical studies are challenging the experimental data in mice and in experiments testing the effect of plasma of patients with FSGS on human podocytes in vitro. It is actually doubtful that suPAR plays a pathophysiological role in human FSGS. Some authors, however, suggest that the different domains of suPAR and the differently glycosylated suPAR molecules might not present the same functional characteristics [56, 65]. Therefore further studies should be initiated using assays discerning the different circulating forms of suPAR in various glomerular pathological conditions [65, 66].

## Some podocyte’s mechanisms possibly targeted by circulating factors in INS

### Podocyte B7-1 (CD80)

The expression of B7-1 (also named CD80) on podocytes in experimental models [67] and in human MCD relapses [30] is associated with the development of proteinuria.

CD80 is a trans-membrane protein expressed on the surface of B cells and other antigen-presenting cells (APC). Toll-like receptor 3 (TLR3) ligands induce CD80 expression in human podocytes via an NF-kappaB-dependent pathway [68]. The expression of podocyte CD80 induced by LPS in vitro through binding to TLR4 is associated with actin reorganization and shape change [67]. Viral products might also stimulate CD80 expression on podocytes. The incubation in vitro of human podocytes with polyIC (polyinosinic-polycytidylic acid, a TLR3 ligand that mimics viral RNA), increases the podocyte expression of TLR3, CD80, and cathepsin L, decreases the expression of synaptopodin, and results in actin reorganization [68]. In vivo polyIC induces proteinuria, glomerular CD80 expression, and increased urinary CD80 in mice [69].

CD80 expression on dendritic cells is inhibited by cytotoxic T-lymphocyte-associated-protein 4 (CTLA-4) and IL-10, which are produced by T regulatory (Treg) cells [70, 71]. CTLA-4 and IL-10 are also produced by podocytes [72].

The expression of CD80 on podocytes, resulting in modifying the podocyte’s cytoskeleton and shape leading to proteinuria, might be a physiological phenomenon aiming to increase the clearance of antigens during infectious or allergic episodes [73]. The pathophysiological hypothesis of a defect in the control of the expression of CD80 has been formulated as follows [72]: under normal circumstances, CD80 expression is only transiently expressed after a triggering event and proteinuria is minimal due to rapid auto-regulatory response by circulating Treg cells or by the podocyte itself, leading to the expression of factors as CTLA-4 that downregulate the podocyte CD80 response [72]. Low circulating CTLA-4 during relapses [74] and the predominance of some CTLA-4 genotypes in INS [75, 76] indirectly support that hypothesis.

CD80 has been proposed to be used for differential diagnosis between MCD and FSGS [59]. Unfortunately, this seems to be difficult in the light of the results presented [59]. Indeed although the mean + − SD of urinary CD80 is higher in MCD in relapse than in FSGS (*p* < 0.001), the mean + − SD of urinary CD80 is higher in FSGS than in controls (*p* = 0.003) and there is an overlap between the lower values of MCD in relapse and the higher values of FSGS [59]. This questions the cutoff value to be used to make this differentiation. Furthermore, the pathophysiological role of CD80 in FSGS is not excluded, since the use of abatacept (CTLA-4-Ig) has been shown to reduce substantially proteinuria in FSGS [77].

### Sphingomyelin phosphodiesterase acid-like 3 b (SMPDL3b)

The discovery that glucocerebrides accumulation in glomerular cells of patients with Gaucher disease resulting in proteinuria raised the interest for the eventual role of sphingolipids in glomerular disease. Sphingolipids are components of the lipid rafts in plasma membranes that are partially associated with nephrin in podocytes. It has been shown that sphingolipid accumulation occurs in several glomerular conditions such as diabetes nephropathy, Lupus nephritis, and FSGS (for a review, see 78). The expression of SMPDL3b, an enzyme that modulates sphingomyelinase activity in podocytes has been shown to be reduced in FSGS (for a review, see 78).

αVβ3 integrin activation occurs in association with decreased podocyte-specific expression of SMPDL3b in kidney biopsy specimens from patients with FSGS. In vitro experiments suggest that the physiological role of SMPDL3b should regulate ß3 integrin activation and prevent podocytes from migration, possibly by interaction with suPAR. Fornoni et al. suggest that targeting SMPDL3b expression in podocyte may prove beneficial in the treatment of FSGS. Those authors have shown that rituximab treatment at the moment of transplantation was associated with lower incidence of post-transplant proteinuria and stabilization of the glomerular filtration rate [79]. In this study, rituximab partially prevented SMPDL-3b down-regulation that was observed in podocytes treated with the sera of patients with recurrent FSGS. Those authors suggest that treatment of high-risk patients with rituximab at the time of kidney transplant might prevent recurrent FSGS by modulating podocyte function in an SMPDL-3b-dependent manner [79].

### Angiopoietin-like 4 (Angptl4)

Initial studies revealed increased podocyte expression of Angptl4 in human and experimental MCD, transient up-regulation after the onset of proteinuria in experimental membranous nephropathy (MN), and no change in podocyte expression in non-HIV collapsing glomerulopathy (CG) and FSGS [80]. Further investigation revealed two types of Angptl4 protein in NS: (a) a hyposialylated form secreted from podocytes in MCD [80, 81]; (b) a neutral p*I* sialylated form of Angptl4 is increased in the circulation of patients with MCD, MN, FSGS, and CG [82]. Most of this sialylated protein is secreted from skeletal muscle, heart, and adipose tissue when proteinuria reaches nephrotic range in an attempt to reduce proteinuria through glomerular endothelial binding; it induces also hypertriglyceridemia via inhibition of lipoprotein lipase (LPL). It is also clear that podocyte-secreted hyposialylated Angptl4 mediates proteinuria in MCD [80, 81]. The effects of hyposialylated Angptl4 are most likely related to its binding to the GBM [80] or to endothelial cells [82]. Those data have led to further studies that reveal that modification of the soluble Angptl4 sialylation or changing key amino acids in its sequence can be successfully used to treat proteinuria [83].

If circulating factors might induce podocyte structure and function abnormalities leading to NS, the primary cause of NS might also be related to an abnormal response of podocytes to common triggering events.

## Conclusions

The attempts to characterize a circulating factor responsible for INS resulted in the identification of several different molecules that can play a role in FSGS or in MCD. However, the primary event remains to be identified and a multifactorial mechanism is probable. Research on the causes of INS has to be continued with several aims: (1) to distinguish at an early stage SSNS from SRNS to avoid useless and toxic steroid therapy; (2) to detect FSGS relapses after transplantation at time in order to start plasma exchange before the formation of definitive glomerular lesions; (3) to set up new treatments aimed to antagonize or to prevent the secretion of the causal agent; (4) future studies should also focus on intrinsic podocytes features which should make the cells more vulnerable to external triggers.

Experimental and clinical studies should be done in the frame of an international multicenter network to avoid possible bias related to the technique used and the selection of patients and controls.

## Key points

1. The pathophysiological role of (a) circulating factor(s) in MCD and FSGS has been suggested by numerous clinical and experimental observations.
2. T and B cells are highly suspected to play a key role in the pathophysiology of MCD- and FSGS-associated nephrotic syndrome.
3. Several molecules have been shown to be able to modify the shape and the proprieties of podocytes and to provoke proteinuria in experimental conditions.
  Indirect evidence exists to suspect the role of some of the latter molecules in the pathophysiology of MCD- and FSGS, however none of them has been identified as the unique primary cause.
4. The pathophysiology of MCD and FSGS probably results from different mechanisms, which could be multifactorial for both diseases.
5. A primary intrinsic dysregulation of the podocyte’s metabolism rending the cell more sensible to external triggers has been advocated recently.
6. FSGS recurrence after transplantation may be prevented by pre-emptive plasma exchange. Recurrence of the disease after transplantation may be successfully treated by plasma exchange when applied early.

## Questions (answers are provided following the reference list)

1. A relapse of nephrotic syndrome after kidney transplantation is:
2. never observed in case of SRNS related to a mutation of a gene coding for a podocyte’ s protein.
3. observed in all cases of FSGS not related with a mutation of a gene coding for a podocyte’s protein.
4. often observed in case of FSGS not related with a mutation of a gene coding for a podocyte’s protein and never observed when such a mutation is present.
5. often observed in case of FSGS not related with a mutation of a gene coding for a podocyte’s protein and rarely observed when such a mutation is present.
6. The molecular weight of the focal sclerosis permeability factor (FSPF) is situated:
7. between 30 and 50 kDa
8. between 50 and 180 kDa
9. above 180 kDa
10. under 30 kDa
11. Recurrence of massive proteinuria after renal transplant for SRNS and FSGS:
12. may be prevented by pre-emptive plasma exchange
13. is not reversible even when treated early by plasma exchange
14. is always reversible under rituximab treatment
15. is reversible after plasma exchange even when histological lesions are already present
16. The soluble urokinase-type plasminogen activator receptor (su-PAR) has been shown:
17. to have a molecular weight superior to the FSPF
18. not to be able to induce proteinuria in mouse
19. to be increased in blood of patients with FSGS only
20. to be consistently correlated with the degree of proteinuria in the diseases studied
21. to be inversely correlated with eGFR and most probably to be the consequence of a reduced clearance
22. What is the parameter which should be able to differentiate with certainty MCD- from FSGS-associated nephrotic syndrome at an early stage?
23. urinary CD80
24. serum suPAR
25. serum suPAR corrected for eGFR
26. serum suPAR/urinary CD80 ratio
27. none of them
